# Radioanalytical Techniques to Quantitatively Assess the Biological Uptake and In Vivo Behavior of Hazardous Substances

**DOI:** 10.3390/molecules25173985

**Published:** 2020-09-01

**Authors:** Jae Young Lee, Sajid Mushtaq, Jung Eun Park, Hee Soon Shin, So-Young Lee, Jongho Jeon

**Affiliations:** 1 Department of Environmental and Safety Engineering, Ajou University, Suwon 16499, Korea; jaeylee@ajou.ac.kr; 2 Department of Nuclear Engineering, Pakistan Institute of Engineering and Applied Sciences, Islamabad 45650, Pakistan; sajidmushtaq@pieas.edu.pk; 3 Department of Applied Chemistry, School of Applied Chemical Engineering, Kyungpook National University, Daegu 41566, Korea; pje1204@knu.ac.kr; 4 Division of Functional Food Research, Korea Food Research Institute, 245, Nongsaengmyeong-ro, Iseo-myeon, Wanju-gun, Jeollabuk-do 55365, Korea; hsshin@kfri.re.kr (H.S.S.); sylee09@kfri.re.kr (S.-Y.L.); 5 Food Biotechnology Program, University of Science and Technology, Daejeon 34113, Korea

**Keywords:** hazardous substances, biodistribution, radiolabeling, in vivo imaging, environmental health

## Abstract

Concern about environmental exposure to hazardous substances has grown over the past several decades, because these substances have adverse effects on human health. Methods used to monitor the biological uptake of hazardous substances and their spatiotemporal behavior in vivo must be accurate and reliable. Recent advances in radiolabeling chemistry and radioanalytical methodologies have facilitated the quantitative analysis of toxic substances, and whole-body imaging can be achieved using nuclear imaging instruments. Herein, we review recent literature on the radioanalytical methods used to study the biological distribution, changes in the uptake and accumulation of hazardous substances, including industrial chemicals, nanomaterials, and microorganisms. We begin with an overview of the radioisotopes used to prepare radiotracers for in vivo experiments. We then summarize the results of molecular imaging studies involving radiolabeled toxins and their quantitative assessment. We conclude the review with perspectives on the use of radioanalytical methods for future environmental research.

## 1. Introduction

Hazardous substances are found in many consumer products. These substances can be harmful to humans, and most of them have not been assessed for toxicity [1,2]. Recently developed nanomaterials can readily enter the environment. They are potentially hazardous to humans when they are inhaled, absorbed, or ingested. Previous studies on hazardous material exposure have investigated how these materials harm humans in both environmental and epidemiological contexts [3,4]. Folletti et al. reviewed studies that found correlations between chemicals in household products and respiratory problems [3]. Witorsch and Thomas [5] critically examined the literature on endocrine disruption by chemicals in personal care products. Needham and Sexton [6] reviewed studies on childhood exposure to hazardous chemicals in the environment. The impacts of hazardous chemicals on health are often investigated by performing experiments on animals under laboratory conditions [4 and references therein]. In this review, we summarize 113 reports from animal exposure studies that have identified strong correlations between chemicals that are commonly found in the environment and have detrimental reproductive, neurological, behavioral, metabolic, and immunologic effects. However, studies involving large human and/or animal populations have been limited. This is because it is difficult to determine the distributions and cycling lifetimes of hazardous chemicals in living organisms. 

To evaluate the impacts of toxic substances on human health, it is essential to assess their biodistribution, changes in uptake, and excretion profile in animal models. However, quantifying the biological uptake of toxic substances is difficult due to the challenges associated with detecting them in tissues, which contain complex biomolecules and chemical species. Mass spectrometers are conventionally used to detect target analytes in biological samples, but they are unsuitable for analyzing macromolecular mixtures with wide-size distributions. Chromatographic methods that employ UV–visible detectors cannot be used to measure analytes in biological 

Unless the analytes generate convenient signals in the UV–visible region. Structural analysis via Raman and Fourier-transform infrared spectroscopy can be performed to identify specific materials in biological samples, but the accuracy of these techniques is limited when multiple carbonaceous substances are present in the sample. Optical imaging with a fluorescent label can be performed to semiquantitatively determine the biodistribution of a molecule or detect trace quantities in vivo [7,8]. However, the amount of quantitative information that can be obtained from deep tissues using this technique is intrinsically limited. This is because optical signals do not penetrate tissues deeply, and photobleaching or quenching of the fluorescent dye can occur. These limitations can be circumvented using radiolabels and radioanalytical methods to accurately assess exposure to toxic substances. A radiotracer is a molecule in which one or more atoms have been replaced by a radioisotope. The radioisotope labeled in a molecule emits energy in the form of radiation, and thus monitoring of radiation decay makes it possible to trace the molecule of interest in biological systems. The use of a well-established radioanalytical technique and an appropriate radiotracer enables the straightforward, accurate, and sensitive detection of target molecules in biological tissues and living subjects [9,10]. Among several types of radioactive decay, gamma (γ) rays are highly penetrating, and they do not interfere with the analytical signal. Noninvasive imaging techniques, such as positron emission tomography (PET) and single-photon emission computed tomography (SPECT), enables the visualization and quantification of target analytes in live subjects [11,12].

In this review, we focus on the recent application of radiolabeling chemistry and radioanalytical methods to assess the biological uptake of hazardous substances. We begin by introducing the most frequently used radioisotopes with a focus on their physicochemical properties and labeling reactions. We then review literature from radioisotopic labeling studies conducted after 2010. Radioisotopic labeling is an important tool for accurate biodistribution determinations and goes beyond the scope of other traditional methods. Next, we discuss the limitations of radioanalytical methods and their prospects in the fields of environmental science and toxicology. Several recent reviews have focused on the development of radiolabeled molecules for medical purposes, such as the diagnosis and treatment of disease [13,14,15,16,17]. In this review, we aim to provide a deeper understanding of radiolabeled materials in the context of tracking hazardous substances and determining their distributions in animal models. We hope this will help extend the application of radiolabeled materials to study the biodistribution of hazardous materials and their effects on the human health. 

## 2. Radioisotopes Used to Determine the Biodistributions of Toxic Materials

**Scheme 1:**
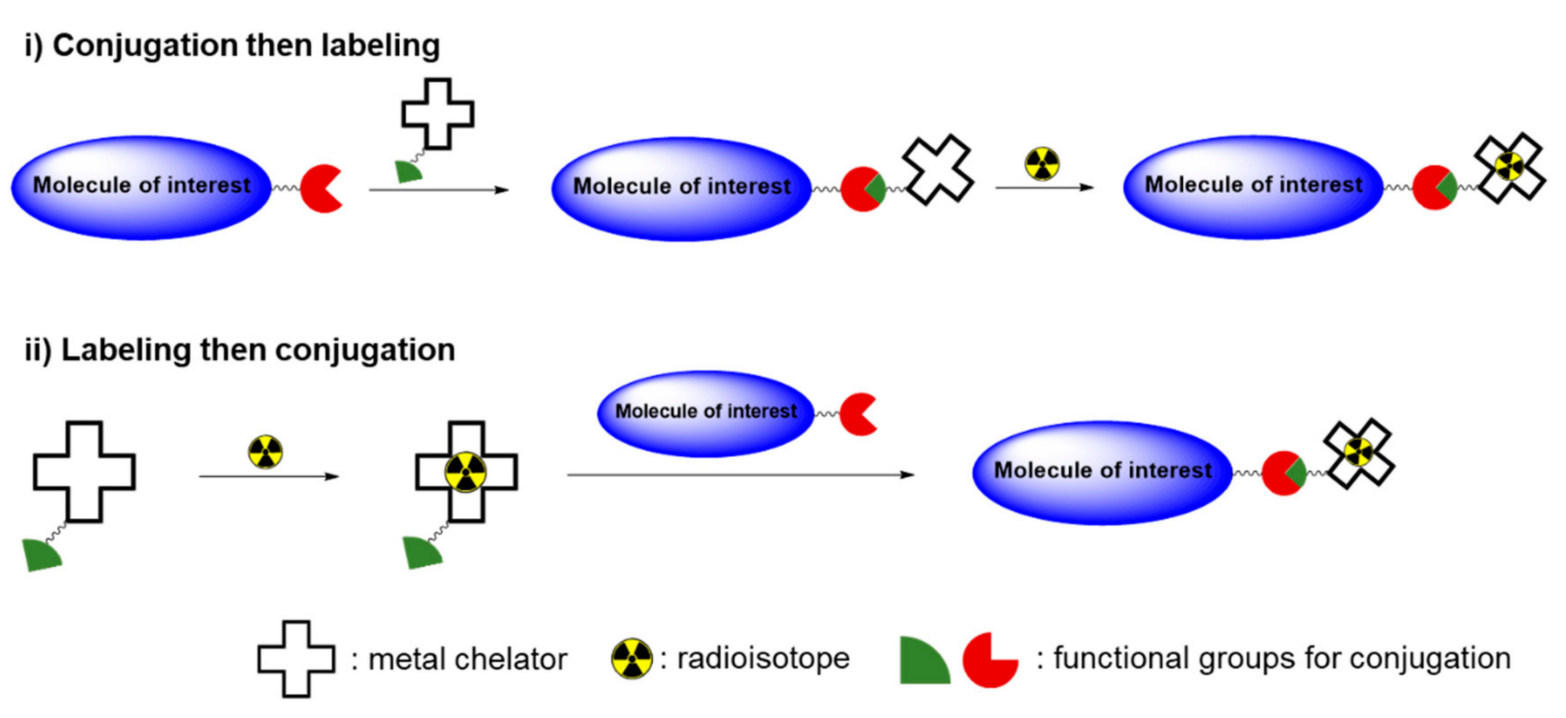
General procedure for labeling of metal radioisotope.

**Scheme 2:**
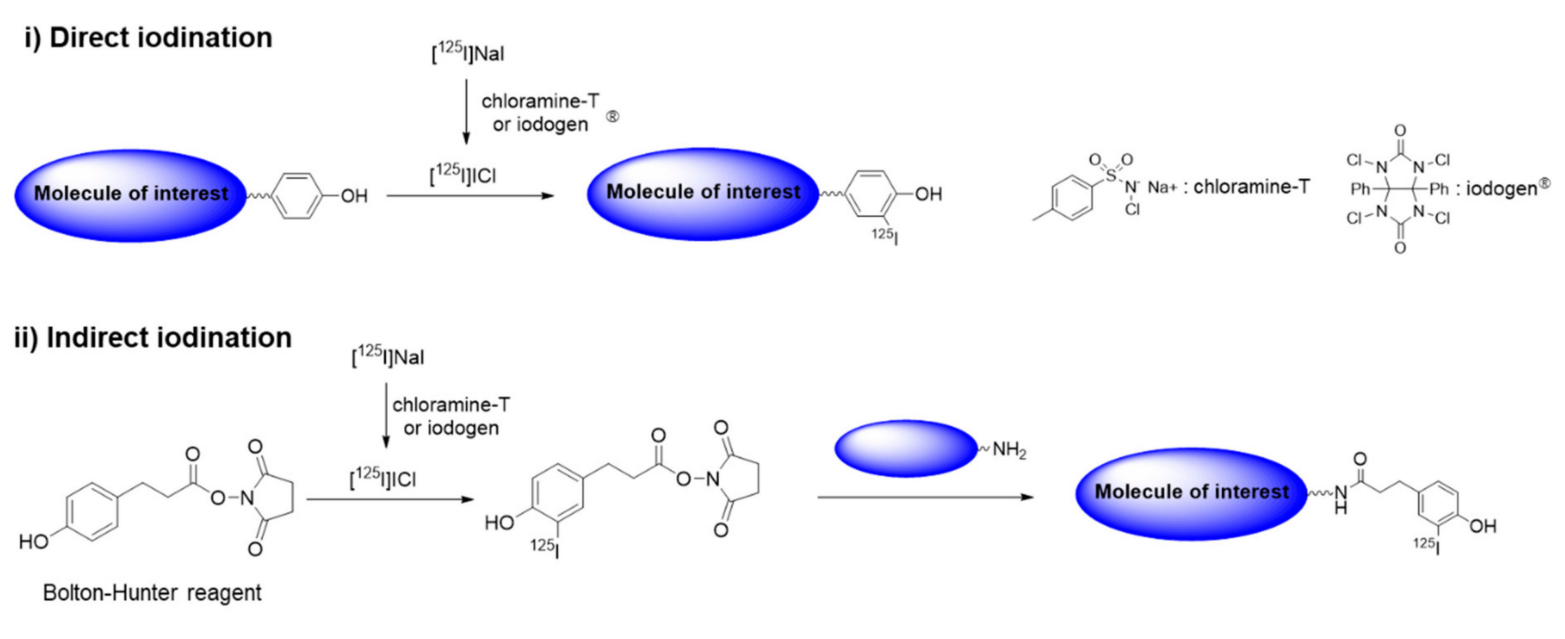
General procedure for labeling of radioactive iodine.

Table 1 is a list of radioisotopes that are commonly used in biodistribution studies along with their physical properties and radiolabeling methods. Carbon-14 (^14^C, *t*_1/2_ = 5730 y) decays to stable nitrogen-14 by emitting a β particle (β^−^) with an energy (*E_β_*) of 0.156 MeV. ^14^C is among the most frequently used radioisotopes along with (^3^H), because the structures of ^14^C-labeled radiotracers are identical to their parent structures. ^14^C-labeled compounds in various chemical classes, including drug candidates, industrial chemicals, and biocides, have been introduced in the last several decades. These compounds are used in pharmacokinetic and pharmacological research to obtain quantitative information about the dispositions of chemicals in organisms. Radioactivity in biological samples can be measured accurately using a liquid scintillation counter (LSC) after pretreatment using a well-established procedure. Fluorine-18 (^18^F, *t*_1/2_ = 109.8 min) is generated in a cyclotron, and it is an important positron (β^+^) emitter for the early and accurate diagnosis of various diseases. Several well-established reactions, such as nucleophilic and electrophilic substitutions, allow the introduction of ^18^F into the molecule of interest. A few ^18^F-labeled compounds are used extensively for in vivo PET imaging [18], although their short half-lives generally make them unsuitable for long-term assessments of hazardous chemicals in live subjects. However, the ^18^F labeling strategy can be used to evaluate toxic chemicals that contain fluorine, including perfluorinated alkyl compounds. Sulfur-35 (^35^S, *t*_1/2_ = 87.3 d) is another β^−^ emitter that is used to produce sulfur-containing radiotracers. Metal radioisotopes, including ^64^Cu, ^99m^Tc, and ^111^In, can be incorporated into target molecules for in vivo tracking and imaging experiments. In general, labeling with a metal radioisotope is performed in two steps. In the first step, a suitable bifunctional chelator, which contains both a metal binding moiety and chemically reactive functional group, is conjugated with the target molecule. The metal radioisotope is then chelated by the chelator attached to the target analyte [19]. Reversely, the bifunctional chelator is firstly labeled with a metal radioisotope, and then the radiolabeled chelator can be conjugated with the molecule of interest (Scheme 1). Metal radioisotopes normally provide better labeling efficiencies than nonmetal radioisotopes; thus, these radioactive metals are quite useful for evaluating macromolecules such as large polymers and biomolecules in vivo. However, changing the structure of a target analyte by incorporating a chelator may affect the biodistribution of the radiolabeled molecules. Some radionuclides (e.g., ^64^Cu, ^111^In) can be labeled as metal nanoparticles by incorporating them into the material core. The chelator-free technique provided highly stable radiotracers that have been applied to in vivo imaging and biodistribution studies [20,21]. Decay emission after electron capture (EC) or an isomeric transition (IT) can be detected using a γ counter and visualized via SPECT. Iodine radioisotopes are important for a variety of medical diagnostic and treatment procedures [22]. Several radioactive iodine isotopes have specific half-lives and decay modes that are quite useful for therapeutic applications (^131^I) and nuclear imaging techniques, including SPECT (^123^I) and PET (^124^I). Iodine-125 (^125^I) is a long-lived isotope (*t*_1/2_ = 59.4 d) that can be used for a kinetics analysis and the quantification of tissue concentrations in biodistribution studies. ^125^I enables a highly sensitive detection with negligible interference from components in the sample matrix. Simply counting the low-energy gamma emission at 35 keV is sufficient for an accurate quantification and imaging of small animals in preclinical research. Radioactive iodine reacts readily with phenolic residues, such as the phenolic moiety of tyrosine, via electrophilic substitution, which requires an oxidizing agent (e.g., chloramine-T, iodogen^®^) (direct iodination, Scheme 2). In some cases, indirect iodination reaction can also be applied to the substrates that lack a phenolic residue or show a low stability under an oxidation condition. In this method, a prosthetic group, such as Bolton–Hunter reagent, is firstly reacted with a radioisotope and then the iodinated product is conjugated with the target molecule.

## 3. In Vivo Assessment of Hazardous Substances Using Radioanalytical Methods

**Figure 1:**
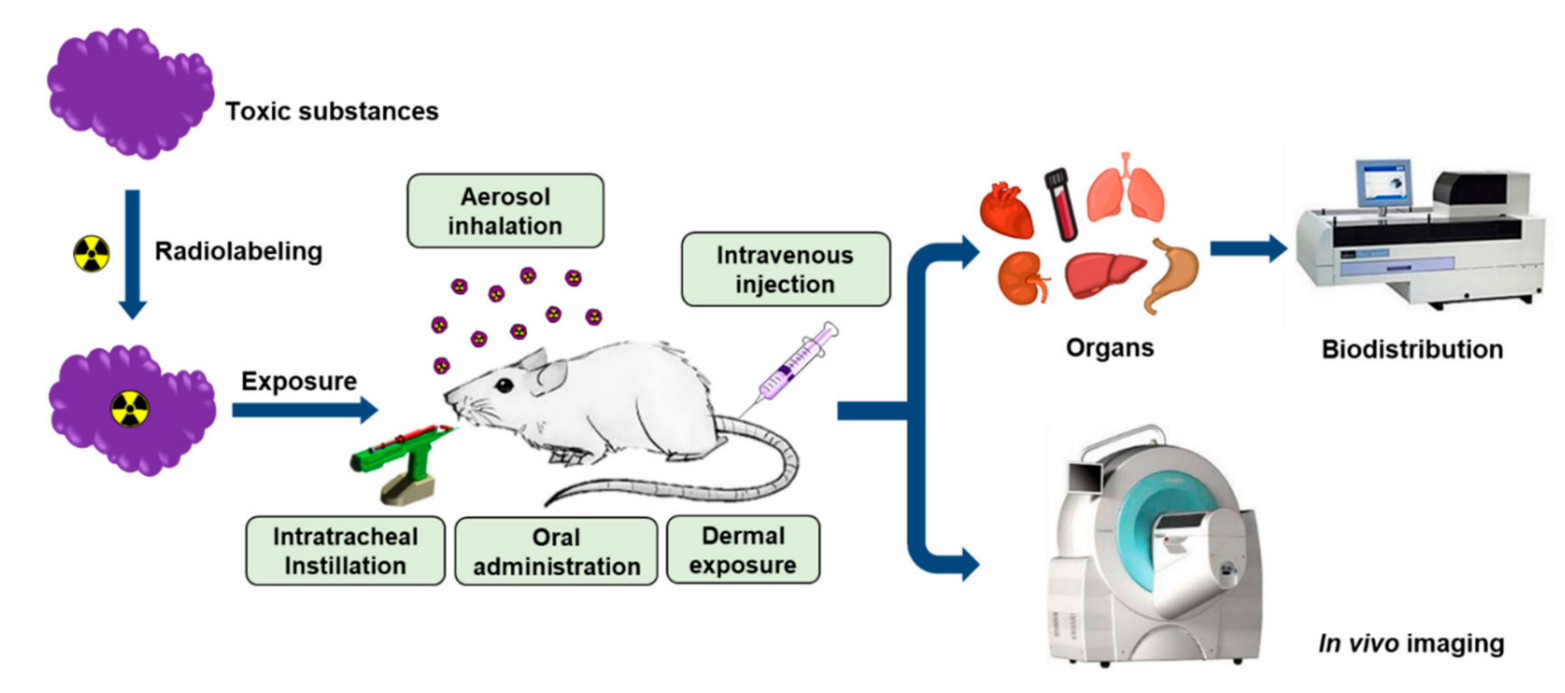
General strategy for in vivo assessment of hazardous substances in animal models.

The general strategy for the quantitative assessment of hazardous substances in animal models is illustrated in Figure 1. The target analyte is first labeled with a suitable radioisotope using an established protocol. Radiolabeled compounds should be stable in living organisms, so the stability of a radiolabeled compound must be evaluated under physiological conditions before it is used for in vivo experiments. The radiotracer is then introduced into the animal model via aerosol inhalation, intratracheal instillation, oral administration, intravenous injection, dermal exposure, or a combination of these routes. To investigate the fate of aerosolized particles in live animals, researchers often utilize specially designed inhalation systems equipped with aerosol particle generators. Exposure chambers can be customized for whole-body or nose-only exposures. At specified times, the animals are sacrificed to harvest their organs and blood for subsequent radioactivity measurements. A gamma counter is used to measure the radioactivity in the collected tissues due to the emission of γ-rays and positrons, and an LSC can be used to measure β^−^ emission. The distribution in each tissue is generally expressed as a percentage of the injected dose (%ID) or as a normalized value relative to the mass of tissue (%ID/g). SPECT or PET scans are utilized to detect the radioactivity emitted from a radioisotope in the preclinical imaging and the resulting images provide spatial and quantitative information about the target analyte in vivo. 

### 3.1. Small Molecules 

The biological uptake and behavior of chemical substances must be assessed in vivo to determine their tolerance thresholds, adverse effects, and mechanisms of action. This information is necessary to identify adverse effects due to the accumulation of chemical substances in tissues or organs. Quantitative data are also useful for determining how exposure to hazardous chemicals causes organ-specific toxicity. Radiolabeled compounds have been utilized in animal studies to perform accurate chemical assessments in vivo. Small structural changes to a low-molecular-weight organic compound during radiolabeling will alter the biological uptake and distribution of the compound. Therefore, the structure of a radiolabeled tracer must be identical to that of the parent compound to ensure that biodistribution analyses using animal models are accurate and robust.

Here we introduce a selection of small molecules developed for radioanalytical methods. Perfluoroalkyl substances (PFASs) are a widely utilized class of industrial organic compounds [23]. PFASs have recently been classified as environmentally persistent organic pollutants of emerging concern, because most of them are resistant to chemical hydrolysis and biodegradation [24]. Some PFASs have been detected in the serum and tissues of humans. The bioaccumulation of PFASs in living organisms can cause several adverse effects [25]. Radiolabeling has been utilized to measure the biological uptake and distribution of organic pollutants in living organisms. Lupton et al. used ^14^C-labeled perfluorooctanoic acid (^14^C-PFOA) to investigate the bioaccumulation of toxic substances in Angus cows [26]. ^14^C-PFOA was orally administered to the cows to determine its pharmacokinetic parameters. Radioactivity in the blood, urine, and feces of the cows was measured for 28 days via LSC or liquid chromatography–tandem mass spectrometry (LC-MS/MS). The cows were sacrificed 28 days after ^14^C-PFOA administration, and the ^14^C-PFOA in tissues was quantified using a radioanalytical method. The results showed that the administered ^14^C-PFOA was excreted in urine within nine days, and no radioactivity was detected in edible tissue. The results of this study suggested that human consumption of meat from cattle exposed to PFOA might not be hazardous. Sundström et al. radiolabeled perfluorooctane sulfonate (PFOS) and perfluorobutane sulfonate (PFBS) with ^35^S to facilitate the evaluation of these chemicals. ^35^S was incorporated via the nucleophilic addition of perfluoroalkyl magnesium chloride in situ to obtain [^35^S]SO_2_ [27]. Animal models were exposed to radiolabeled PFOS to investigate the distribution of PFOS in their organs [28] and a placental transfer of the compound [29] using LSC and whole-body autoradiography. After oral exposure to the substrate, PFOS was distributed to most tissues in a dose-dependent manner. Large accumulations were observed in several internal organs, including the liver, lungs, kidneys, and bones [28]. In 2017, Lapi et al. reported labeling three perfluorinated alkyl substances (PFAS) with ^18^F [30]. To prepare the radiotracers without modifying their structures, they performed an isotopic exchange (^19^F → ^18^F) via the nucleophilic substitution of the native compounds. The three ^18^F-labeled PFAS, which contained different alkyl groups, were intravenously administered to normal mice. A biodistribution analysis revealed that perfluorooctanoic acid and perfluorohexanoic acid were highly concentrated in the liver. The uptake of the perfluorobutanoic acid analog, which had a shorter alkyl chain, was the highest in the stomach. Although ^18^F-labeled tracers are not useful for studying excretion kinetics, they could be used to measure the uptake by human subjects more readily than other radiotracers due to the wide availability and short radioactive half-life of ^18^F.

Bisphenol A (BPA) is an important monomeric compound that is used to manufacture a wide range of industrial products, including polycarbonate plastics and resins. BPA is used to manufacture a large proportion of the synthetic chemicals produced worldwide, and an estimated 4 × 10^6^ tons of BPA-derived chemicals were produced in 2015 [31]. Toxicological studies have indicated that BPA can enter the human body through several pathways, including digestive, respiratory, and dermal routes [32]. BPA exposure has been shown to induce carcinogenesis and mutagenesis in animal models. BPA is also thought to be capable of causing multiorgan toxicity and genotoxicity [33], and recent data indicate that high BPA levels are associated with the development of several types of tumor cells [34]. Consequently, BPA uptake and pharmacokinetics have been investigated in various animal models using ^14^C-labeled and ^3^H-labeled BPA tracers. In vivo BPA exposure through several possible routes, including intravenous injection [35], intraperitoneal injection [36], and dermal exposure, has been assessed in vivo [37]. The application of radiolabeled BPA in biodistribution studies is summarized in Table 2.

The previously described radioanalytical methods could also be applied to monitor trace elements. Lapi et al. exposed animal models to a metal radioisotope to investigate the biological distribution of trace minerals [38]. They produced a positron-emitting manganese radionuclide (^52^Mn) from chromium via proton bombardment. Manganese is an essential micronutrient. However, the prolonged uptake of large amounts of manganese can cause neurotoxicity and lead to severe neurological pathology. Therefore, measuring trace quantities of manganese and determining its in vivo biodistribution are necessary to better understand its toxicity and wider biomedical relevance. Mn(II) has been utilized as a contrast agent for magnetic resonance imaging. An aqueous solution of [^52^Mn]MnCl_2_ was injected intravenously into mice in an in vivo study. In a separate experiment, animals in a closed chamber were exposed to aerosolized [^52^Mn]MnCl_2_. The ex vivo biodistribution of radioactivity indicated that ^52^Mn was distributed throughout the body, and high concentrations were observed in several internal organs, including the lungs, liver, kidneys, and thyroid gland. The results of both experiments confirmed that manganese crossed the blood–brain barrier. The quantitative measurement of Mn in living subjects can provide useful information about its toxicity, which can be used to compose informed safety guidelines for environmental and occupational manganese exposure.

### 3.2. Nanomaterial

In this section, we discuss the use of radioanalytical methods for in vivo assessments of nanomaterials. Rapid developments in nanotechnology over the past several decades have expanded the range of available nanomaterials. This expansion has been accompanied by growing concern about the potential health effects of these materials [51]. The number of applications for functional nanomaterials in the biomedical field has also increased, and some of them are useful for the diagnosis and treatment of disease [52]. However, the bioaccumulation, pharmacokinetics, and potential toxicity of a novel nanomaterial should be evaluated before it is mass-produced or approved for clinical use.

The unique physical and chemical properties of two-dimensional graphene and graphene oxide-based nanocomposites have garnered significant attention from researchers in areas ranging from basic science to applied engineering [53]. These materials have been used to produce many different industrial chemicals for over a decade. Functionalized graphene and graphene oxide derivatives in particular have demonstrated potential for a variety of biomedical applications, including biosensing, molecular imaging, tissue engineering, and drug delivery [54]. It is essential to investigate the environmental safety of these materials before they are extensively employed for such applications. Several researchers have reported toxicity in animal models based on the biodistribution of graphene nanomaterials. Yang et al. were the first to report the long-term distribution of functionalized nanographene in vivo [39,55]. A nanographene sheet (NGS) modified with polyethylene glycol was radiolabeled with ^125^I using chloramine T as an oxidant. Radioactive iodine atoms were bound to the phenyl rings in graphene via electrophilic aromatic substitution. The radiolabeled product was injected intravenously, and its pharmacokinetics were investigated by determining its blood and organ distribution on day 1 and day 60, respectively. The results suggested that an NGS accumulated primarily in the liver (16% ID/g) and spleen (25% ID/g) within one hour of injection. It was then excreted via the gastrointestinal and urinary routes, although clearance was quite slow. An NGS had no obvious toxic effects on mice injected with a dose of 20 mg/kg for three months.

In the field of toxicology, inhalation is the most concerning route of exposure to nanomaterials. This is because nanomaterials have been found to deposit primarily in lung tissue. Li et al. performed radioisotope tracing of nanoscale graphene oxide (NGO) in mice after the carbon-based nanomaterial was labeled with ^125^I. After purifying the radiolabeled product, it was administered to Kunming mice via an intratracheal instillation to monitor its uptake in vivo [40]. The biodistribution data collected 10 min after exposure showed that a significant amount of ^125^I-labeled NGO was retained in the lungs (70.3% ID). The radioactivity level decreased to 20.2% ID within 12 h of exposure. A small amount of the NGO may have translocated to other organs, including the intestines, via the air–blood barrier in the respiratory system. SPECT images of the ^125^I-labeled NGO in the same animal models also indicated that the material was strongly retained by the lungs. In contrast to the results of a previous toxicity study in which NGO was introduced via an intravenous injection, intratracheally instilled NGO could cause acute lung injury and chronic pulmonary fibrosis. Guo et al. incorporated ^14^C into graphene for the first time to investigate the biodistribution of a carbon-based nanomaterial in a live subject [41]. The radiolabeled product was prepared by adding ^14^C-labeled phenol to graphene nanosheets after graphitization at high temperatures. No byproducts were detected after the labeling procedure, which indicated that the ^14^C was successfully incorporated into the graphene. Small planktonic crustaceans (*Daphnia magna*) were then exposed to water that had been spiked with the ^14^C-labeled graphene to quantify the bioaccumulation of the nanosheets. Depuration of the graphene by *Daphnia magna* depended strongly on the presence of chemical and biological additives in the water. In a subsequent study, the researchers utilized the same labeling method to investigate the biological uptake of few-layer graphene (FLG) and its inhalation toxicity using ICR mice [42]. In the biological uptake experiment, 46.2% of the intratracheally instilled FLG was excreted in the feces. Nearly half of the FLG (47%) was found remaining in the lungs four weeks after exposure. Exposure to unlabeled FLG induced acute lung injury and pulmonary edema in a dose-dependent manner. Measurements of radioactivity in other tissues indicated that a small amount of ^14^C-labeled graphene translocated from the lungs to other organs. A negligible amount of FLG was absorbed through the gastrointestinal tract following oral administration. Although ^14^C-labeled FLG cannot be used for nuclear imaging, it is more useful for long-term biodistribution studies than ^125^I-labeled graphene derivatives. In addition, ^14^C-labeled tracers can be used to analyze metabolites and excretion. In general, the labeling procedure of radioactive iodine is significantly simpler than that of ^14^C labeling. However, it is often difficult to accurately quantify ^125^I-labeled analogs in animal models due to the low in vivo stability of the C-I bond and high accumulation of radioactive iodine in the thyroid [56,57,58,59,60]. 

In 2017, Smith et al. reported the results a systematic in-depth study on the long-term clearance and translocation patterns of inhaled aerosols [43]. They used 10, 15, 35, and 75 nm radioactive iridium (^192^Ir) nanoparticles (NPs) as model materials and exposed rats to aerosolized NPs using a nasal exposure system. The whole-body distribution of radioactivity and the distributions of radioactivity in the organs and excreta were monitored for several months. The quantity of ^192^Ir in the biosamples and animals indicated that the inhaled NPs had long retention half-lives (100–500 d) in the lungs. Low levels of translocated radioactivity were observed in secondary target organs, including the kidneys and liver, and smaller particles accumulated to a greater extent. Although iridium is not an emerging toxin, the methodology and results of this study are relevant for the investigation into the biodistribution of inhaled nano-aerosols.

### 3.3. Macromolecules

Hazardous chemicals are present not only in industrial products and synthetic materials, they are often found in products designed for household use. For example, some household products contain polyhexamethylene guanidine phosphate (PHMG). PHMG is a serious health risk, because its toxicity has not been evaluated thoroughly. Measuring the accumulation of PHMG in the body is essential for an organ-specific toxicological analysis. However, it is difficult to quantify its distribution in biological tissues using typical analytical instrumentation. In 2018, our research group reported using a novel radioanalytical technique to determine the biodistribution of PHMG aerosols [44]. We first labeled polymeric PHMG with ^111^In. The ^111^In-labeled PHMG was then aerosolized to an average size of 270 nm and delivered to a whole-body animal exposure chamber. Radioactivity was monitored via radio thin-layer chromatography and radio HPLC. High levels of PHMG were detected in the lungs 0.5 h after exposure, and we found that approximately 74% of the PHMG was retained 168 h later. In addition, a portion of the PHMG translocated to the liver. These observations indicated that significant respiratory uptake of PHMG and its slow clearance could cause fatal inhalation toxicity in humans. This was the first study in which the biological uptake of a seriously harmful aerosolized organic polymer was quantified.

**Figure 2:**
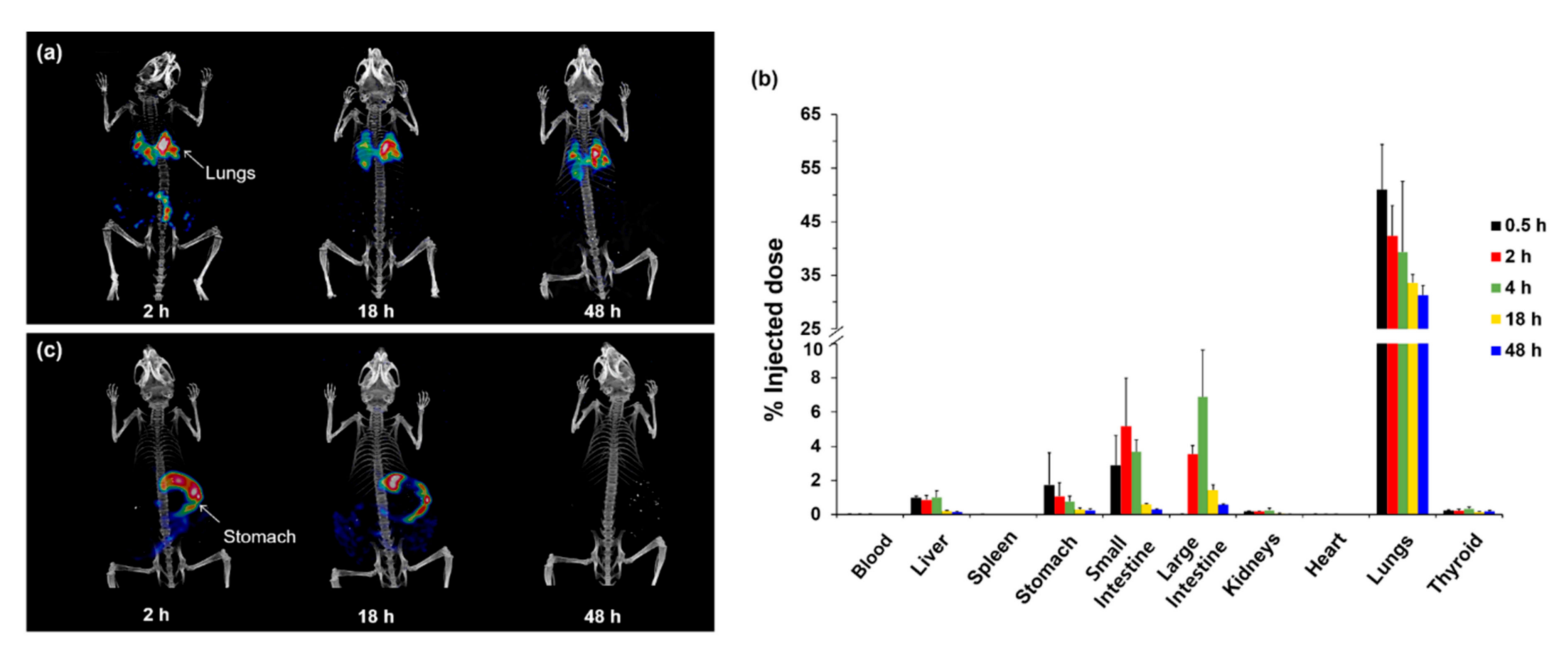
(**a**) Whole-body single-photon emission computed tomography (SPECT)/CT images of mice exposed to radiolabeled diesel exhaust particulates (^125^I-DEP) via intratracheal instillation. (**b**) Biological distribution of intratracheally instilled ^125^I-DEP. (**c**) Whole-body SPECT/CT images of mice after oral administration of ^125^I-DEP. Reprinted with permission from Lee et al. [45].

Fine particulate matter (PM) in the air has recently been recognized as an emerging pollutant in many counties. Among the various types of PM, particulates in diesel exhaust particulates (DEP) emitted by diesel-fueled engines are known to have adverse effects on human health [61]. Toxicological studies have reported that continual exposure to DEP may cause several respiratory diseases. DEP has been classified as a Group 1 human carcinogen by the International Agency for Research on Cancer [62]. DEP is made up of polycyclic aromatic hydrocarbons (PAHs), and the particles range in size from <100 nm to the submicron scale. The organic particles do not generate any detectable signal in live subjects, but radiolabeling and nuclear imaging techniques can be used to quantitatively assess DEP in vivo. Our group labeled pyrene with radioactive iodine, and it was efficiently incorporated into PAH agglomerates [45]. Normal mice were then exposed to the radiolabeled DEP (^125^I-DEP), which ranged in size from 70 to 800 nm, via intratracheal instillation. The SPECT/CT images and biodistribution results indicated a high degree of DEP accumulation in the lungs and slow clearance. Forty-eight hours after exposure, only 30% of the DEP initially taken up by the lungs was excreted or translocated to other internal organs (Figure 2). Interestingly, orally administered ^125^I-DEP was quickly cleared from the body, and no radioactivity was detected 48 h after exposure. These results highlighted the value of testing different exposure routes to assess the in vivo behavior of DEP. We expect that this approach can be extended to the analysis of other hazardous PM and synthetic chemicals.

Lipopolysaccharide (LPS) molecules are considered endotoxins, and they are components of the outer membranes of Gram-negative bacteria. An LPS macromolecule consists of a hydrophilic core, a polysaccharide chain, a hydrophilic *O*-antigenic polysaccharide side chain, and hydrophilic lipid A. Hydrophilic lipid A is responsible for the toxic properties of LPSs. Exposure to LPS molecules induces a strong immune response in human hosts, which leads to severe adverse effects [63]. To understand the uptake and tissue distribution of LPS in vivo, Denat et al. developed a dual labeling method and utilized SPECT/CT and fluorescence microscopy for detection [46]. They synthesized a trifunctional molecular probe that contained BODIPY, DOTA, and isothiocyanate. After the probe was linked to LPS, the DOTA-BODIPY-LPS conjugate was labeled with ^111^In. SPECT/CT images showed that the injected LPS was rapidly distributed to the liver and spleen, and significant amounts were retained for at least 24 h. Florescence imaging of excised liver samples indicated the hepatic uptake of LPS, which was consistent with the in vivo imaging results and the organ distribution ex vivo. The authors demonstrated that the use of a radiolabeling technique for whole-body and cellular imaging was more effective than previously reported radiolabeling approaches, and they achieved a kinetic analysis of LPS administered to live subjects. 

### 3.4. Microorganisms

**Figure 3:**
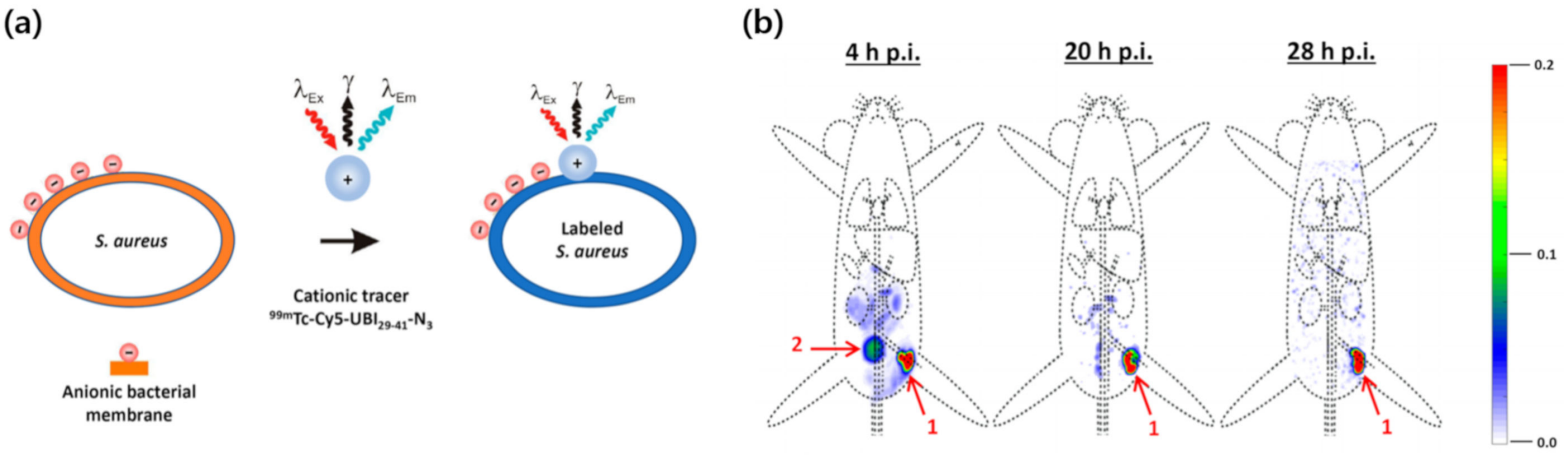
(**a**) Radiolabeling of *S. aureus* with a ^99m^Tc-UBI29-41-Cy5 peptide probe. (**b**) SPECT images of radiolabeled *S. aureus* in a mouse after inoculation in a thigh muscle. Organs are marked as (1) inoculation site and (2) bladder. Reprinted with permission from Welling et al. [48].

For years, the tracking and characterization of microorganisms in vivo has attracted considerable attention in studies on diagnostic tools and therapeutic drugs for serious infections [64]. Many countries are battling infectious microorganisms, including SARS-CoV-2, the virus that causes COVID-19. Thus, there is a growing demand for analytical tools to facilitate noninvasive monitoring of microbial distributions and dissemination in vivo. A wide range of genetically modified bacteria and viruses have been investigated for potential applications in disease therapy, and methods to accurately visualize administered microorganisms can be useful biomedical tools [65]. Radiolabeling methods have been employed to track bacteria and viruses in animal models in vivo. Technetium tricarbonyl [^99m^Tc(CO)_3_] has a high affinity for hexahistidine (His6) residues in proteins. In 2018, Jeon et al. developed a convenient ^99m^Tc labeling method to study *Escherichia coli* (*E. coli*) under mild physiological conditions [47]. A genetically engineered vector was used to express an artificial protein in the bacterial cells that contained multiple His6 sequences, which made it possible to label the protein with ^99m^Tc(CO)_3_. ^99m^Tc(CO)_3_ was incubated with the cells at 37 °C (pH = 7.5) to afford radiolabeled *E. coli*. SPECT/CT imaging and biodistribution study make it possible to analyze the initial uptake values and changes in accumulation of both orally administered and intravenously injected *E. coli*. In a subsequent study, Welling et al. achieved multimodal tracking of *Staphylococcus aureus* (*S. aureus*) in a mouse model [48]. They labeled an antimicrobial peptide with ^99m^Tc and Cy5 (^99m^Tc-UBI29-41-Cy5) for use as a tracer. The positively charged ^99m^Tc-UBI29-41-Cy5 tracer efficiently bound to the negatively charged bacterial membrane. Radiolabeled *S. aureus* was then injected locally into thigh muscles to investigate the translocation of the infectious bacteria (Figure 3). Biodistribution analysis was performed 28 h after injection, and the signal had decreased by only 15% at the inoculation site. Some of the *S. aureus* cells that accumulated in the muscle were excreted via the renal clearance route. ^99m^Tc enabled facile radiolabeling and a clear visualization of the administered bacterial cells. Further optimization would allow the range of applications for this method to be expanded. However, it is not practical to use ^99m^Tc for long-term in vivo imaging due to its short half-life (6 h). To overcome this limitation, radioactive rhenium (^188^Re, ^186^Re) can be used instead of ^99m^Tc. The coordination chemistry of these radioisotopes is quite similar to that of ^99m^Tc [66], and they have longer half-lives of 16.9 h (^188^Re) and 89.2 h (^188^Re). The signal intensities of these radioisotopes do not depend on the viability of bacterial cells, so nuclear imaging can be coupled with bioluminescence imaging using a reporter gene to monitor live bacteria. 

**Figure 4:**
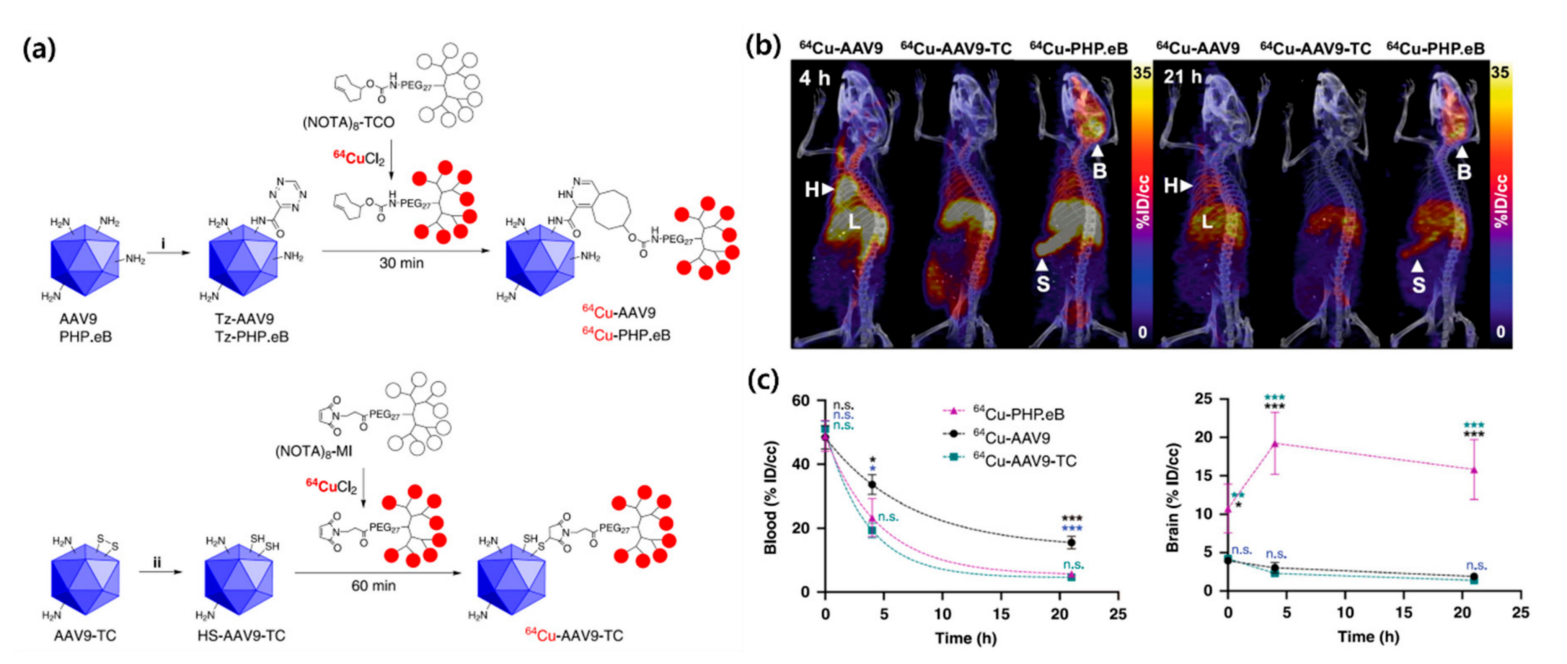
(**a**) Radiolabeling of adeno-associated viruse (AAV) capsids. (**b**) PET/CT images of ^64^Cu-labeled AAV 4 h and 21 h after intravenous injection (B = Brain, H = Heart, L = Liver, S = Spleen). (**c**) Time–activity curves for blood and brain uptake. Reprinted with permission from Seo et al. [50].

Several studies have employed radiolabeled viral particles, engineered adeno-associated viruses (AAVs) in particular. These viruses have 25 nm protein capsids that encapsulate single-stranded deoxyribonucleic acid, and they have been investigated as efficient vehicles for therapeutic delivery to the brain [67]. Thus, in vivo imaging and biodistribution measurements can be used to develop optimized AAVs. Kothari et al. tracked the distribution of AAV vectors in vivo by performing noninvasive imaging [49]. To accomplish this, they labeled AAV serotype 10 capsids with the positron emitter ^124^I using direct and indirect radiolabeling methods. In the direct radiolabeling reaction, tyrosine residues in the viral capsids were directly iodinated. The authors used an ^124^I-labeled acylation agent for the indirect method. The radiolabeled AAV vectors were administered to mice via an intracranial injection, and in vivo PET/CT scanning was performed to study their changes in the uptake in the brain for eight days. Although the images clearly show the spatial and temporal distributions of the viral vectors with minimal deiodination, administration of the radiolabeled AAV vectors was limited to a direct infusion into the brain. Seo et al. recently developed a highly efficient AAV radiolabeling method [50]. They synthesized unique, multichelating prosthetic groups that bore specific functional groups for rapid *trans*-cyclooctene-tetrazine or maleimide-thiol bioconjugation with engineered AAV9, AA9-TC, and AAV-PHP.eB capsids (Figure 4). An exposed loop on the surface of the engineered AAV-PHP.eB capsid contained a 2-mer substitution and a 7-mer peptide insertion. After labeling each prosthetic group with ^64^Cu, it was reacted with a surface-modified viral vector to obtain radiolabeled AAV (^64^Cu-AAV) with high molar activity (μCi/pmol). PET imaging and a biodistribution analysis of intravenously injected ^64^Cu-AAV indicated that the engineered virus rapidly accumulated in the brain. The observed uptake values for the AAV-PHP.eB capsid were higher than those of the other AAVs. The affinity of the AAV-PHP.eB capsid for the brain was thus higher than that of the well-known AAC9 capsid. These results suggest that radiolabeling and PET imaging studies can be useful tools to monitor the binding affinity and pharmacokinetics of viral particles. Similar radiolabeling methods and experimental designs can be applied to assess other types of infectious viruses.

## 4. Conclusions and Future Perspectives

In this review, we provided an overview of recent studies in which radioanalytical techniques were employed to assess hazardous substances in animal models. We focused on the preparation of radiolabeled materials, the quantitative analysis of biological uptake, and bioimaging applications. Radiotracer methods have notable advantages over other methods in terms of accuracy and sensitivity, and radioactive signals are unaffected by biological tissues and complex biomolecules. Owing to these advantages, the bioaccumulation and behavior of hazardous and potentially toxic substances have been investigated using radioanalytical methods. We expect that radioanalytical methods will be employed for the quantitative in vivo assessment of emerging contaminants, including a wide range of inhaled fine PM and ingested submicron plastics. Many radionuclides are commercially available; thus, routes of exposure for hazardous trace elements, such as heavy metals, in air or water can be investigated in vivo. It is necessary to develop more efficient radiolabeling procedures that can provide high radiochemical results and in vivo stability along with suitable exposure systems for animal models to broaden the range of radioanalytical applications. It should be noted that radioanalytical methods may not be suitable for the identification of toxin metabolites, because metabolites may form derivatives that no longer contain the radioisotope tag or that possess different chemical and biological properties. To avoid this issue, metabolic analysis using radiotracers should thus be coupled with other sensitive detection techniques. Moreover, the partial structural changes caused by radiolabeling procedures (e.g., conjugation of chelating agent) may affect the biodistribution profile of the molecule of interest. Nevertheless, radioisotopic analyses will be useful for determining exposure routes, tracing pollutants and toxicants in the body, and for risk assessments. This is because radioanalytical methods have been shown to be effective in measuring target analytes that are difficult to detect using conventional analytical tools. We hope this review article will provide in-depth insight into radioanalytical methods to inspire more active research and encourage the development of new applications. This will ultimately contribute to the accurate and reliable assessment of the biological uptake and toxicological effects of substances that are hazardous to human health and the environment.

## Figures and Tables

**Table 1:** Physical properties of radioisotopes used to study the biodistributions of toxic substances.

Radioisotope	Decay Half-Life	Decay Mode	Detection Instrument(s)	Radiolabeling Method
**^14^** **C**	5730 y	β^−^	LSC ^1^	Incorporation into an organic molecule
**^18^** **F**	109.8 min	β^+^	γ counter, PET ^2^ (imaging)	Nucleophilic or electrophilic substitution
**^35^** **S**	87.3 d	β^−^	LSC	Addition reaction, metabolic labeling (for amino acids)
**^52^** **Mn**	5.6 d	β^+^	γ counter, PET (imaging)	Chelation
**^64^** **Cu**	12.7 h	β^+^, β^−^, EC ^3^	γ counter, PET (imaging)	Chelation
**^99m^** **Tc**	6.0 h	IT ^4^	γ counter, SPECT ^5^ (imaging)	Chelation
**^111^** **In**	2.80 d	EC	γ counter, SPECT (imaging)	Chelation
**^123^** **I**	13.2 d	EC	γ counter, SPECT (imaging)	Electrophilic substitution
**^124^** **I**	4.18 d	β^+^, EC	γ counter, PET (imaging)	Electrophilic substitution
**^125^** **I**	59.4 d	EC	γ counter, SPECT (imaging)	Electrophilic substitution
**^192^** **Ir**	73.8 d	β^−^, EC	γ radiography	-

^1^ Liquid scintillation counting; ^2^ positron emission tomography; ^3^ electron capture; ^4^ isomeric transition; ^5^ single-photon emission computed tomography.

**Table 2:** In vivo assessments of selected toxic substances.

Substance	Radioisotope and Labeling Method	Exposure Route	Animal Model	Results	Ref.
Perfluorooctanoic acid	^14^C, incorporation	Oral exposure	Angus cows	Biodistribution of ^14^C-labeled perfluorooctanoic acid in blood, urine, and feces over 28 days and in tissues at the time of slaughter (28 days) Measurement of plasma elimination half-life	[26]
Perfluorooctane sulfonate	^35^S, nucleophilic addition to [^35^S]SO_2_	Oral exposure	C57BL/6 mice	Biodistribution of low and high doses of ^35^S-labeled perfluorooctane sulfonate in organs over 5 d Whole-body autoradiogram of a mouse 48 h after oral administration	[28]
	^35^S, nucleophilic addition to [^35^S]SO_2_	Gestational exposure	Pregnant C57BL/6 mice	Biodistribution of ^35^S-labeled perfluorooctane sulfonate in the organs of dams, fetuses, and pups Whole-body autoradiograms of pregnant mice 48 h and 96 h after exposure	[29]
Perfluorinated alkyl compounds	^18^F, isotopic exchange (^19^F → ^18^F)	Intravenous injection	CD1 mice	Biodistribution of ^18^F-labeled perfluorocarbons in organs over 4 h Comparison of biological uptake values of three different perfluorocarbons (PFOA, PFHxA, PFBA)	[30]
Bisphenol A	^14^C, incorporation	Intravenous and topical injection, percutaneous absorption	SD rats (in vivo), human skin (in vitro)	Quantification of radioactivity in urine, feces, skin, and carcasses (^14^C-BPA mass balance) 1–72 h after administration In vivo and ex vivo measurement of percutaneous ^14^C-BPA absorption	[35]
	^14^C, incorporation	Intraperitoneal injection	Pregnant mice	Biodistribution of ^14^C-BPA in the organs of pregnant mice and newborn mice determined via autoradiography	[36]
	^14^C, incorporation	Percutaneous absorption	Human skin (in vitro)	Kinetic measurements of ^14^C-BPA penetration Distribution of ^14^C-BPA in the stratum corneum after incubation for 24 h	[37]
Manganese	^52^Mn	Nasal inhalation, intravenous injection	CD1 mice	Biodistribution of inhaled and injected ^52^Mn in organs over 24 h Whole-body PET/CT imaging of intravenously injected ^52^Mn over 72 h	[38]
Graphene	^125^I, electrophilic substitution	Intravenous injection	BALB/c mice	Biodistribution of ^125^I-labeled graphene in organs over 60 days Hepatic and renal toxicity over a three-month period after exposure	[39]
Graphene oxide	^125^I, electrophilic substitution	Intratracheal instillation	Kunming mice	Biodistribution of ^125^I-labeled graphene oxide in organs over 12 h Whole-body SPECT/CT imaging for 1 h Pulmonary toxicity over a three-month period after exposure	[40]
Graphene	^14^C, graphitization with ^14^C-labeled phenol	Intake	*Daphnia magna*	Quantification of ^14^C-labeled graphene uptake and excretion in different aqueous media	[41]
Graphene	^14^C, graphitization with ^14^C-labeled phenol	Intratracheal instillation, oral exposure	ICR mice	Biodistribution of ^14^C-labeled graphene in organs over 3 d (oral exposure) or 28 d (intratracheal instillation) Quantification of translocation from the lungs to other internal organs Pulmonary toxicity 24 h after exposure	[42]
Iridium NPs	^192^Ir, incorporation	Nasal inhalation	SD rats	Measurement of radioactive iridium NP (10, 15, 35, and 75 nm) retention half-life in the lungs Quantification of translocation from the lungs to other internal organs	[43]
PHMG	^111^In, DOTA chelation	Whole-body inhalation, intratracheal instillation	SD rats	Biodistribution of ^111^In-labeled PHMG aerosols in organs over 168 h Quantification of translocation from the lungs to other internal organs Whole-body SPECT/CT imaging over 24 h	[44]
DEP	^125^I, self-assembly with ^125^I-labeled pyrene	Intratracheal instillation, oral exposure	ICR mice	Biodistribution of ^125^I-labeled DEP particles over 48 h Whole-body SPECT/CT imaging over 48 h	[45]
LPS	^111^In, DOTA-BODIPY conjugate chelation	Intravenous injection	C57BL/6 mice	Biodistribution and SPECT/CT imaging of ^111^In-labeled LPS in organs over 24 h Ex vivo fluorescence imaging of liver tissues	[46]
*E. Coli*	^99m^Tc, [^99m^Tc(CO)_3_] chelation with intracellular proteins	Intravenous injection, oral exposure	ICR mice	Organ biodistribution of ^99m^Tc-labeled *E. Coli* over 8 h Whole-body SPECT/CT imaging over 8 h	[47]
*S. Aureus*	^99m^Tc, radiolabeled peptide attached to the cell membrane	Intramuscular injection	Swiss mice	Organ biodistribution of ^99m^Tc-labeled *S. Aureus* over 28 h Whole-body SPECT and fluorescence imaging of infected muscle over 28 h	[48]
AAV	^124^I, electrophilic substitution/modified Bolton–Hunter reagent	Intraparenchymal injection	CD-1 mice	Whole-body PET/CT imaging of ^124^I-labeled AAV over 8 h days Measurement of brain uptake over 8 d	[49]
AAV	^64^Cu, NOTA chelation followed by conjugation on the AAV surface	Intravenous injection	C57BL/6 mice	Organ biodistribution of ^64^Cu-labeled AAV over 21 h Whole-body PET/CT imaging over 21 h Comparison of AAV9, AA9-TC, and PHP.eB viral capsid uptake by the brain	[50]

## Abbreviations

PET	Positron emission tomography
SPECT	Single-photon emission computed tomography
CT	Computed tomography
LSC	Liquid scintillation counter
ID	Injected dose
PFASs	Perfluoroalkyl substances
PFOA	Perfluorooctanoic acid
LC-MS/MS	Liquid chromatography–tandem mass spectrometry
PFOS	Perfluorooctane sulfonate
PFBS	Perfluorobutane sulfonate
BPA	Bisphenol A
NGS	Nanographene sheet
NGO	Nanoscale graphene oxide
FLG	Few-layer graphene
PHMG	Polyhexamethylene guanidine phosphate
LPS	Lipopolysaccharide
DOTA	1,4,7,10-Tetraazacyclododecane-1,4,7,10-tetraacetic acid
BODIPY	4,4-difluoro-4-bora-3a,4a-diaza-*s*-indacene
AAVs	Adeno-associated viruses
